# Temporal Trends and Recent Correlates in Sedentary Behaviors among Chinese Adults from 2002 to 2010–2012

**DOI:** 10.3390/ijerph17010158

**Published:** 2019-12-24

**Authors:** Caicui Ding, Ganyu Feng, Fan Yuan, Weiyan Gong, Yecheng Yao, Yanning Ma, Yan Zhang, Ailing Liu

**Affiliations:** Department of Nutrition and Health Education, National Institute for Nutrition and Health, Chinese Center for Disease Control and Prevention, Beijing 100050, China; dingcc@ninh.chinacdc.cn (C.D.); fenggy@ninh.chinacdc.cn (G.F.); yuanfan@ninh.chinacdc.cn (F.Y.); gongwy@ninh.chinacdc.cn (W.G.); yaoyc@ninh.chinacdc.cn (Y.Y.); mayn@ninh.chinacdc.cn (Y.M.); zhangyan@ninh.chinacdc.cn (Y.Z.)

**Keywords:** sedentary behaviors, trends, correlates, China, adults

## Abstract

Evidence suggests that more time spent in sedentary behaviors (SB) increases health risk independent of physical activities. Trends in SB among adults have not been fully described in China, and the sociodemographic correlates of SB have not been systematically evaluated either. This study examined the temporal trends of SB among 184,257 adults (2002: *n* = 52,697, 2010–2012: *n* = 131,560) using data from the China National Nutrition and Health Survey in 2002 and 2010–2012, and analyzed the recent correlates of SB in Chinese adults. Overall, an increase (+0.29 h/d) was seen in total SB across the survey years, and there was a slight increase (+0.14 h/d) in leisure time SB and a decrease (−0.39 h/d) in occupational SB. From 2002 to 2012, the proportion of Chinese adults whose total SB time over 4 h/d increased from 35.4% to 43.0%, and the proportion of leisure SB time over 3 h/d increased from 42.0% to 48.0%, and the proportion of occupational SB time over 4 h/d decreased from 63.4% to 53.0%. Male, urban areas, employed, unmarried, and with higher educational and family economic level were all positively associated with high sedentary time (HST) in 2010–2012. These trends and correlates are important for health policy in China and other countries that are facing similar challenges.

## 1. Introduction

Physical inactivity is the fourth highest risk factor for death in the world [1]. However, due to the advancements of technology, the growing affordability of washing machines, motorized vehicles, and smart phones, many physical activities (PA) have been replaced by sedentary behaviors (SB) during work and leisure time. SB are becoming of higher concern; one reason is that SB may displace the time available for PA and result in overall lower energy expenditure, the other is that SB are related to the risk of many chronic health outcomes independent of PA. Evidence suggests that more time spent in SB increases the risk of all-cause mortality, cardiovascular disease mortality, cardiovascular disease, type 2 diabetes, and cancer of the colon, endometrium, and lung [2].

Over the last few decades, China has experienced significant social and economic transitions, and alongside this transition is the dramatic increase in the prevalence of overweight, obesity, and chronic non-communicable diseases (NCDs). Coupled with China’s rapidly aging population, NCDs contributed to a significant proportion of deaths and rising health care costs in recent years [3]. Understanding trends of NCDs’ factors is critical to developing preventions and interventions. One reason for NCDs is the dramatic increase in energy intake from animal-source food and edible oils [4]; another is the decrease in PA [5] due to the decline in PA intensity and duration and the replacement of SB.

SB have been defined as a range of endeavors with an energy expenditure ≤1.5 times the resting energy expenditure [6]. SB include using a computer or pad, watching TV, reading/writing/drawing, watching videos, playing games, or chatting/talking with friends on the phone while sitting during leisure time and/or at work [7,8]. In view of the lack of evidence, there is not a quantitative key guideline for SB time over the world.

There is fewer data on temporal trends in SB among adults when compared to PA. There were studies from the USA [9,10,11], Australia [12,13], Mexico [14], Denmark [15], and parts of China [16,17]. Most studies indicated an increasing trend in SB, but the increase values and rates were different. China is a typical example of the rapidly developing countries. Analysis of China’s national trends in SB and integration of international comparisons will provide important information to the world.

The objectives of the study were to explore the temporal trends and correlates of SB among adults using data from the China National Nutrition and Health Survey (CNNHS) in 2002 and 2010–2012. The study will provide the epidemic situation of SB in China and the key population of SB, which is critical for future programs aiming to reduce SB in China and the world.

## 2. Materials and Methods

### 2.1. Study Design

The CNNHS was a nationally representative cross-sectional study conducted by the National Institute for Nutrition and Health, Chinese Center for Disease Control and Prevention (NINH, China CDC) to assess the health and nutrition of Chinese civilians, which was the largest and most comprehensive study of nutrition and health outcomes ever in China. The 2002 and 2010–2012 survey covered all 31 provinces, autonomous regions, and municipalities directly under the central government throughout China (except for Taiwan, Hong Kong, and Macao). The design and detailed methods of CNNHS 2002 and 2010–2012 have been described previously [18,19,20]. Briefly, the country was divided into six strata (large cities, small to medium cities, class 1 rural areas, class 2 rural areas, class 3 rural areas, and class 4 rural areas) in 2002 and four strata (large cities, small- and medium-sized cities, general rural areas, and poor rural areas) in 2010–2012 according to their administrative division, population, and level of economic development. The participants were recruited using a stratified multistage cluster sampling design in 2002 and a stratified multistage cluster and probability proportional to size (PPS) sampling design in 2010–2012. Ethics approval was obtained from the Ethics Committee of China CDC in 2002 and NINH, China CDC in 2010–2012 (2013–018). All participants gave informed consent.

### 2.2. Data Collection

**Figure 1:**
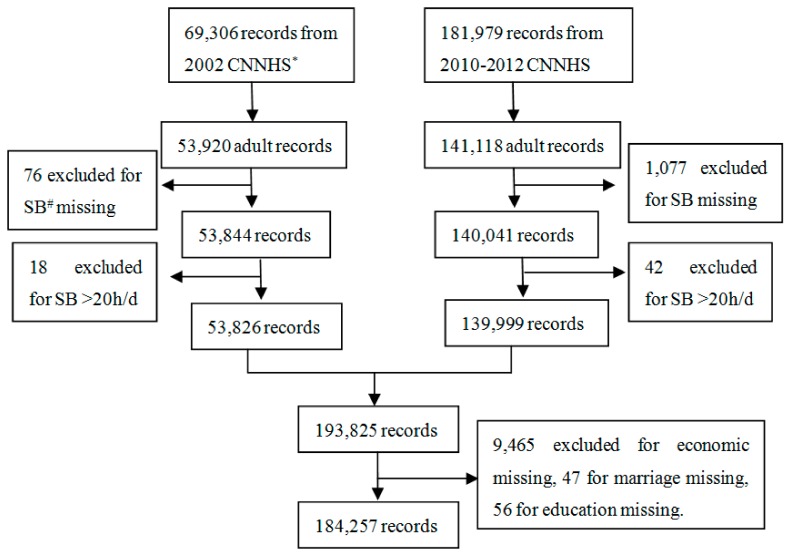
The flowchart of data cleansing. (^*^ CNNHS: China National Nutrition and Health Survey; ^#^ SB: sedentary behaviors)

A face-to-face interview using standard questionnaires was conducted at the home of participants. Similar survey questionnaires were conducted in the two surveys. The General Questionnaire included demographic information. Information on SB was included in the one-year Physical Activity Questionnaire, which included questions about time and intensity of work, transportation, domestic and leisure-time physical activity, time of occupational, leisure time sedentary behaviors, and sleeping. The one-year Physical Activity Questionnaire used in the CNNHS was evaluated to be valid for Chinese population [21]. The total number of respondents to the Physical Activity Questionnaire in CNNHS 2002 and 2010–2012 was 251,285. There were 195,038 participants aged 18 years or older included in the analysis. The participants who had missing responses for the measured activities and the demographic (marriage status, educational level and family economic level) data were excluded. The participants whose SB time was over 20 h/d were also excluded, as sleep time was believed to be at least 4 h/d. In total, 184,257 (2002: *n* = 52,697 [52.6% female], 2010–2012: *n* = 131,560 [55.7% female]) adults with complete data for all SB variables and covariates were included (Figure 1).

### 2.3. Sedentary Behaviors (SB)

In this analysis, SB were divided into occupational SB and leisure time SB. For occupational SB, participants were asked their sitting time during work in a typical weekday. For leisure time SB, they were asked the time spent in watching TV, using computer, reading, playing cards, playing video games, and other SB in leisure time. Total SB time was summed together and dichotomized as either high sedentary time (HST, ≥4 h/d) or low sedentary time (LST, <4 h/d). This classification approximates an apparent threshold of screen based activity that is associated with increased risks of ill health compared to lower volumes [22]. Considering that leisure time SB are more likely to be consciously reduced relative to occupational SB, we also calculated the proportion of leisure time SB over 3 h/d (high leisure sedentary time, HLST), which was examined to be associated with increased mortality regardless of PA in some studies [22,23]. Because of the lack of quantitative guideline for SB time, the time of total SB, leisure time SB and occupational SB was divided into several levels (<2 h/d, 2–4 h/d, 4–6 h/d and >8h/d for total and occupational SB; <1 h/d, 1–3 h/d, 3–5 h/d and >7 h/d for leisure time SB) and the changes in distribution of them were described, which can be comparable with other studies [10,24].

### 2.4. Sociodemographic Characteristics

Demographic information included the participant’s date of birth, sex, occupation, marital status, educational level, and family annual income. The per capita family annual income in each survey was categorized into five grades according to the income level at the time. In the 2002 survey, the grade 1 to grade 5 of the family economic level referred to <800, 800~1999, 2000~4999, 5000~9999, and ≥10,000 Yuan/Year/per capita, while in the 2010–2012 survey, the grade 1 to grade 5 level referred to <5000, 5000~9999, 10,000~14,999, 15,000~24,999, and ≥25,000 Yuan/Year/per capita. The occupation was categorized into employed, farmers, or unemployed. The employed included those who were employed in non-farming occupations and self-employed shop keepers. The unemployed did neither farming nor employed work, and their main activity was housework.

### 2.5. Statistical Analysis

To provide estimates that were representative of the Chinese population, the study used sampling weights calculated based on China census population [25,26]. As the age and gender distribution of the Chinese population was different between 2002 and 2010–2012, in the analysis of the mean time of SB and the prevalence of HST and HLST, we calculated age and gender standardize values based on the 2010 national census data.

As there were 9465 participants (about 5%) with missing values of the family economic level, a sensitivity analysis was done to see if the group with missing values would affect the results.

Descriptive data were reported as weighted means or weighted proportion for each survey year, stratified by demographic characteristics. Because a threshold for increased risks of sitting has not been established, the weighted medians (P25–P75) of SB time from 2002 to 2010–2012 were also reported. T-tests, variance analysis, and Chi-square tests were performed among groups and survey years.

In the analysis of the recent correlates of SB in the 2010–2012 survey, logistic regression analysis was applied. Stepwise elimination process was applied for model choice; the retained variables were result of the stepwise procedure. Sociodemographic variables, including sex, age group, urban or rural residence, occupation, marital status, educational level, and family economic level, were retained in the model. The analysis using P75 as cut-off were also conducted and compared with the results we reported. The significance level was set at *p* < 0.05 using two-sided tests. All the analyses were conducted using the software package SAS version 9.4 (SAS Institute Inc., Cary, NC, USA).

## 3. Results

### 3.1. Characteristics of Participants

The characteristics of participants are shown in Table 1. Increase in percentage of the participants over 50-year old, urban residence, and unemployed occupation, as well as decrease in percentage of farmers, were seen in both male and female from 2002 to 2010–2012 (Supplementary Materials: Table S1), reflecting the situation of aging and urbanization in China.

### 3.2. Sedentary Behavior Trends

**Figure 2:**
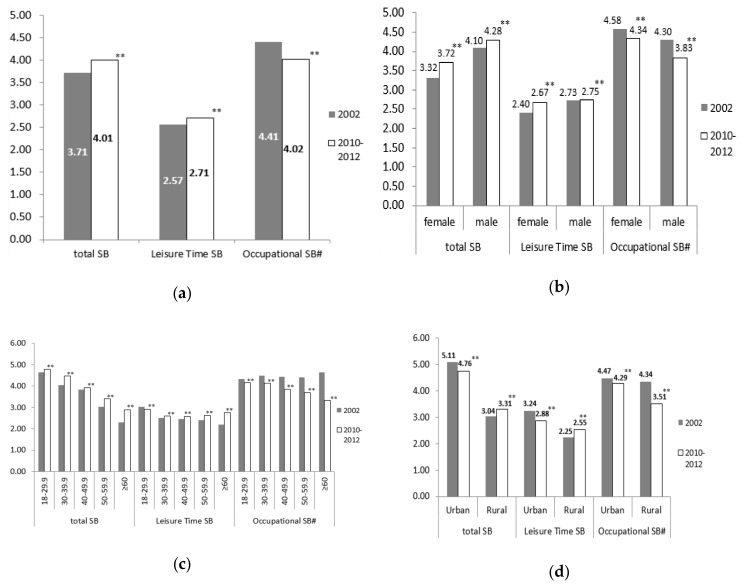

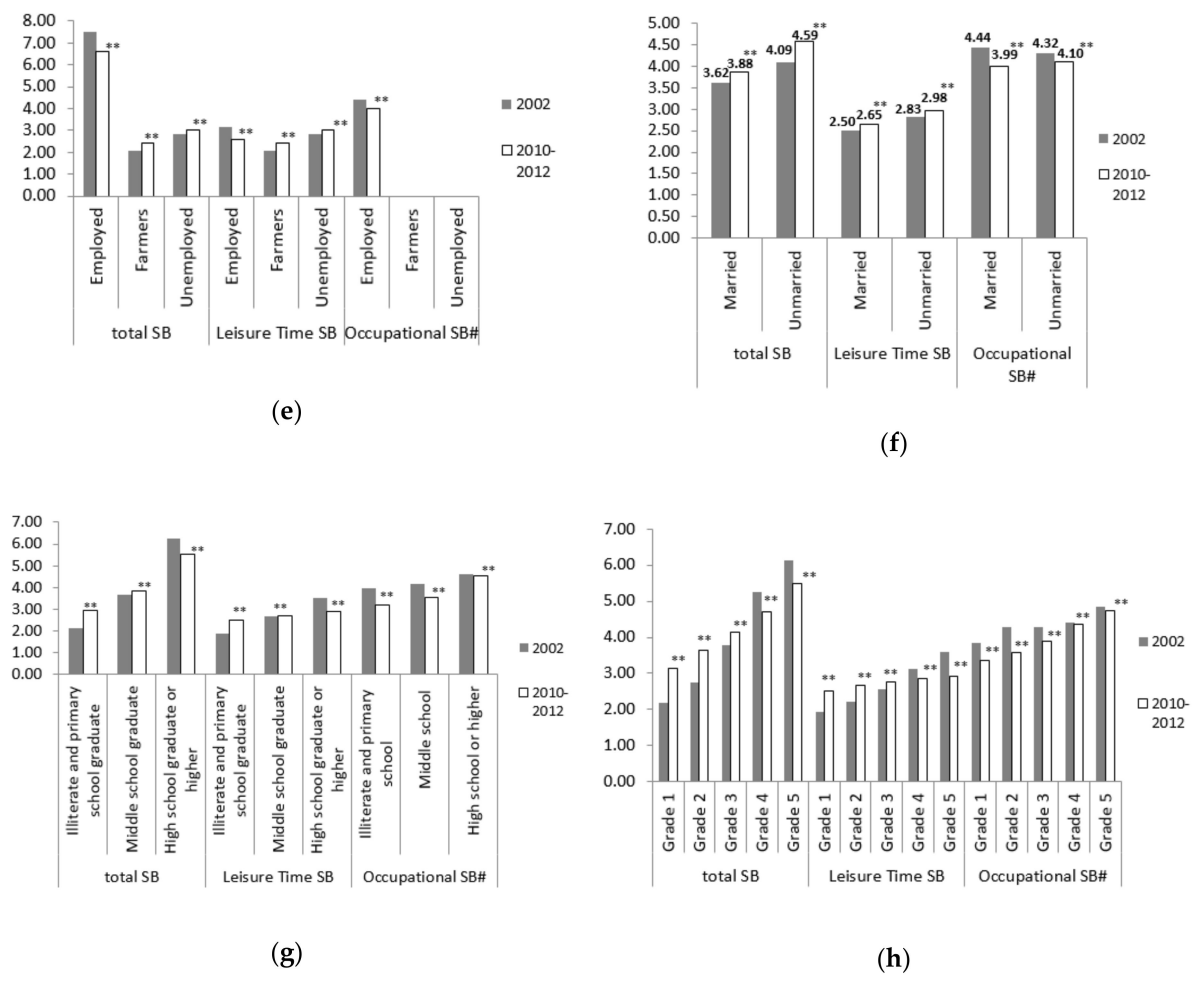
Changes ^§^ in time of sedentary behaviors (SB) from 2002 to 2010–2012 (hours per day): (**a**) changes in total participants; (**b**) changes by gender; (**c**) changes by age group; (**d**) changes by region; (**e**) changes by occupation; (**f**) changes by; (**g**) changes by educational level; (**h**) changes by family economic level (^§^ gender and age standardized to the 2010 China census population; ^#^ Time of Occupational SB was only calculated in employed participants, *n*2002 = 12,625, *n*2010–2012 = 31,914; ^**^ *p* < 0.001, significance is based on T-Tests).

Overall, an increase (+0.29 h/d, *p* < 0.001) was seen in total SB time across the survey years, and there was a slight increase (+0.14 h/d, *p* < 0.001) in leisure time SB and a decrease (−0.39 h/d, *p* < 0.001) in occupational SB (Figure 2(a)). The total SB time reduced by about 1 h among the participants who were employed, reduced by 40 min among those with the highest educational level and the highest family economic level, and by 20 and 30 min among those in urban areas and with a grade 4 family economic level, respectively, while they were increased in other subgroups. There was an obvious decrease in occupational SB time in each subgroup, and more significantly among those who were male, older, in rural areas, with lower educational level, and lower family economic level. The leisure time SB increased in most subgroups except for those who were 18–29.9 years old, in urban areas, employed, with the highest educational level and grade 4 and 5 family economic level (Figure 2(b)–(h)) (Supplementary Materials: Table S2).

There was little change in medians (P25–P75) of SB time among Chinese adults from 2002 to 2010–2012, the total and occupational SB time kept for 3.0 (2.0–5.0) h/d and 4.0 (2.0–6.0) h/d, respectively, and the leisure SB time changed from 2.0 (1.5–3.0) h/d to 2.0 (2.0–3.0) h/d. The total SB time increased in most subgroups, except for those who were in urban areas, employed, with the highest educational level, and grade 4 and 5 family economic level. The occupational SB time decreased in most subgroups (Table 2).

**Figure 3:**
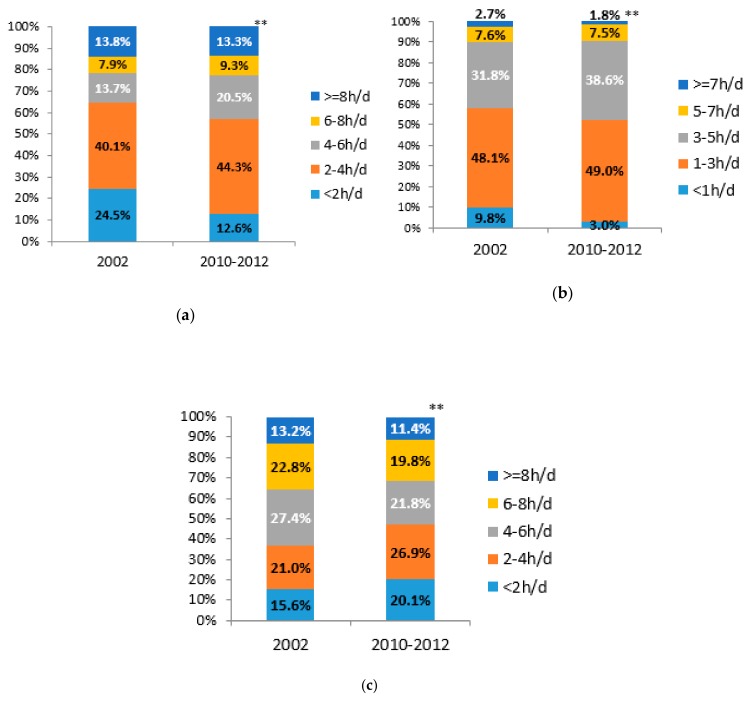
Changes ^§^ in distribution of SB time among Chinese adults from 2002 to 2010–2012 (%): (**a**) changes in total SB time; (**b**) changes in leisure SB time; (**c**) changes in occupational SB time ^#^ (^§^ gender and age standardized to the 2010 China census population; ^#^ Time of occupational SB was only calculated in employed participants, *n*2002 = 126,25, *n*2010–2012 = 31,914; ^**^ *p* < 0.001, significance is based on Chi–square tests).

The weighted prevalence of HST increased from 35.4% to 43.0%, and it increased in most subgroups, except for those who were in urban areas, employed, with the highest educational level, and grade 4 and 5 family economic level. The prevalence of HST was the lowest in farmers among all subgroups, but increased significantly from 2002 to 2010–2012. The HST prevalence in participants with the lowest educational level and the lowest family economic level almost doubled in the 10 years (Table 3). In 2002, the total SB time was more concentrated in <2 h/d and 2–4 h/d; 10 years later, total SB time was more concentrated in 2–4 h/d and 4–6 h/d (Figure 3(a)).

The weighted prevalence of HLST increased from 42.0% to 48.0%, and the trends among subgroups were the same as HST. The prevalence of HST and HLST both increased in the middle school graduate or lower educational level and the grade 1 to grade 3 family economic levels, and the difference value was smaller in higher levels. The prevalence both decreased in the high school graduate or higher educational levels and the grade 4 to grade 5 family economic levels, and the difference value was bigger in higher levels (Table 3). The leisure time SB mainly concentrated in 1–3h/d both in 2002 and 2010–2012, and the proportion within 1 h/d decreased while 3–5 h/d increased (Figure 3(b)).

The prevalence of occupational SB time ≥ 4 h/d decreased from 63.4% to 53.0%, and it decreased in each subgroup. More reductions were among those who were male, older, in rural areas, with lower educational levels and lower family economic levels. (Table 3). In 2002, the occupational SB time was more concentrated in 4–6 h/d and 6–8 h/d; 10 years later, the occupational SB time was more concentrated in 2–4 h/d and 4–6 h/d. Thus, the proportion within 4 h/d increased, while that of ≥4 h/d decreased (Figure 3(c)).

### 3.3. Correlates of Sedentary Behaviors

Table 4 shows the correlates of HST, HLST, and occupational SB time ≥ 4 h/d among Chinese adults in the recent survey years. Male (Odds ratio [OR]: 1.10, 95% confidence intervals [95% CI]: 1.10–1.11), in urban areas (OR: 1.26, 95% CI: 1.26–1.27), employed (OR: 9.56, 95% CI: 9.55–9.56), and unmarried (OR: 1.28, 95% CI: 1.27–1.28) were all positively associated with HST. Participants with higher educational and family economic level were more likely to report HST. Adults aged ≥ 30 years and famers were less likely to report HST compared with those aged 18–29.9 years (OR: 0.68–0.78) and the unemployed (OR: 0.52, 95% CI: 0.52–0.52).

The correlates of HLST were similar to HST, except for occupational factors. Compared with the unemployed, famers (OR: 0.65, 95% CI: 0.65–0.66) and the employed (OR: 0.55, 95% CI: 0.55–0.55) were both less likely to report HLST.

Male (OR: 0.74, 95% CI: 0.74–0.74), aged 40–59.9 years (OR: 0.90–0.95), and unmarried (OR: 0.90, 95%CI: 0.89–0.90) were all negatively associated with occupational SB time ≥ 4 h/d. Participants in urban areas, with higher educational and family economic level were more likely to have the occupational SB time ≥ 4 h/d.

The correlates analysis using P75 as cut-off was conducted. The correlates of total SB time ≥ 5 h/d were similar to that of total SB time ≥ 4 h/d. And the correlates of occupational SB time ≥ 6 h/d were similar to that of occupational SB time ≥ 4 h/d except for age factors. Compared with the 18–29.9 group, participants in older age groups were less likely to report occupational SB time ≥ 6 h/d, the OR (95% CI) was 0.93 (0.92–0.93), 0.85 (0.84–0.85), 0.77 (0.77–0.78), and 0.76 (0.76–0.77), respectively, in 30–39.9, 40–49.9, 50–59.9, and ≥60 groups.

### 3.4. Sensitivity Analysis of the Missing Values

In the sensitivity analysis, the 9465 cases with economic missing were included and marked as economic “unknown”. All the results were recalculated. There was little change in the SB trends among the whole population, and the correlates of SB were similar (Supplementary Materials: Table S3).

## 4. Discussion

The study provides a comprehensive look at SB among Chinese adults for the period 2002–2012. There was a slight increase of total SB time in Chinese adults, which was smaller compared with the US adults during the period of 2007–2016 (from 5.5 h/d to 6.4 h/d) [10,11], and was similar with the adults in Mexico City from 2006 to 2015 (from 3.6 h/d to 3.8 h/d) [14]. The proportion of total SB time over 4 h/d increased by 12.4% among adults in China, while in Queensland Australia the proportion over 3 h/d increased from 40.8% to 46.0% during the period of 2002–2008 [9], and in Mexico City the proportion over 7 h/d increased from 13.7% (2006) to 14.8% (2015) [14]. Overall, total SB time of Chinese adults, which was lower compared with other countries [21,27], showed a smaller increase during the similar periods. It worth noting that the study was conducted during the period of rapid urbanization in China, and with the process of urbanization more SB time may be spent in Chinese adults and need further research.

The occupational SB time among Chinese employed decreased by 0.5 h/d, quite different from Australians, which was stable in 3.8 h/d from 2007 to 2014 [13], and Danes, which increased by 13.2 min per day between 2007 and 2010 [15]. In addition, the total and leisure SB time among Chinese employed decreased by about 1 h/d and 0.5 h/d, respectively, while both were stable among Australians during the period of 2007–2014. The downward trends of SB among Chinese employed may be due to nationwide regulations on working hours in early 21st century, which provided that employees worked 5 days a week (changed from 48–72 h/week to 40 h/week), or their increased health awareness.

This analysis found that the trends of SB in urban and rural China were significantly different. In urban areas, the total and leisure SB time and the prevalence of HST and HLST all decreased, while all increased in rural areas. This may be one reason for the difference of some NCDs’ increase rates between urban and rural areas. From 2002 to 2012, the prevalence of overweight and obesity, hypercholesterolemia, and type 2 diabetes of Chinese adults all increased significantly, and the increase rates in rural China (44.0%, 304.2% and 265.2%) were much higher than in urban areas (20.3%, 197.6% and 108.5%) [28]. Although the causal relationship cannot be proven, it suggested that the SB time among rural residents should be more concerned in the future.

From 2002 to 2010–2012, the total and leisure SB time increased among participants who were in lower educational and economical levels, while they decreased in higher levels. In the 2002 survey, the total SB time increased with the improvement of educational and economical level, and after 10 years, the gap among the levels became smaller. There were similar trends in the study of Mexico City [14], in which the total SB time increased with the improvement of socioeconomic status in 2006, and the gap among the levels became smaller in 2015. The reason may be that with the rapid urbanization, the lifestyles of people with lower socioeconomic levels changed rapidly, but the health consciousness might not have improved accordingly.

Using the recent survey data, the study also found that the correlates of spending more hours on total and leisure time SB were similar to those in other countries [10,29]. HST were strongly associated with male, in urban areas, employed, unmarried, and with higher educational and family economic levels. The reason seems to be that people with higher educational and family economic levels may have more sedentary jobs opportunities, be more likely to use cars, and have more electronic entertainment and labor-saving devices at home [29]. The correlates of SB found in this study could be useful for other developing countries that are undergoing similar transitions to China.

Considering the health risks associated with SB, the Global Action Plan on Physical Activity (2018–2030) for the first time adopted SB reduction as one of the strategies for global chronic disease prevention and control [30]. The second edition of the seminal 10-year update of the Physical Activity Guidelines for Americans had suggested that most people would benefit from both increasing moderate to vigorous physical activity and reducing time spent sitting [31]. The Physical Activity Guideline is being revised in China; this study would be a critical step before population-wide physical activity strategies be developed and implemented.

One of the study’s strength was the utilization of the large, nationally representative surveys with a rigorous protocol and quality control. In addition, the study specifically analyzed the trends of both leisure time SB and total SB time, and explored the potential sociodemographic correlates. One limitation of the study was that self−reported SB may not reflect the true amount of sitting. Secondly, the recent survey did not investigate the leisure SB time of TV, computer, or mobile phone separately, which resulted in a lack of analysis in different domains. Thirdly, the cross–sectional design does not allow for examination of causal relationships when analyzing correlates of HST and HLST. Nevertheless, self-reported time of leisure SB [32], occupational SB, and total SB [33] have been widely used in epidemiological studies, and measurement errors were unlikely to affect findings on the temporal trends [34].

## 5. Conclusions

There was little change in total SB time, a slight increase in leisure time SB, and a decrease in occupational SB among Chinese adults from 2002 to 2010–2012, and the trends had obvious subgroup differences. Male, in urban areas, employed, unmarried, with higher educational and family economic level were all positively associated with HST in 2010–2012. These trends and correlates are important for health policy in China and other countries that are facing similar challenges.

## Figures and Tables

**Table 1:** Characteristics of participants by survey year [*n* (%)].

Variables	2002	2010–2012
Total	52,697 (100.0)	131,560 (100.0)
Gender		
Female	27,737 (52.6)	73,282 (55.7)
Male	24,960 (47.4)	58,278 (44.3)
Age group(year)		
18–29.9	7994 (15.2)	13,820 (10.5)
30–39.9	12,934 (24.5)	18,779 (14.3)
40–49.9	33,654 (22.1)	29,810 (22.7)
50–59.9	10,195 (19.3)	30,898 (23.5)
≥60	9920 (18.8)	38,253 (29.1)
Region		
Urban	17,458 (33.1)	65,219 (49.6)
Rural	35,239 (66.9)	66,341 (50.4)
Occupation		
Employed	12,625 (24.0)	31,914 (24.3)
Farmers	22,801 (43.3)	38,717 (29.4)
Unemployed	17,271 (32.8)	60,929 (46.3)
Marital Status		
Yes	45,889 (87.1)	115,402 (87.7)
No	6808 (12.9)	16,158 (12.3)
Educational Level		
Illiterate and primary school graduate	22,267 (42.3)	54,335 (41.3)
Middle school graduate	18,434 (35.0)	45,767 (34.8)
High school graduate or higher	11,996 (22.7)	31,458 (23.9)
Family economic level		
Grade 1	7562 (14.3)	35,498 (27.0)
Grade 2	15,681 (29.8)	32,381 (24.6)
Grade 3	15,295 (29.0)	25,225 (19.2)
Grade 4	9050 (17.2)	23,925 (18.2)
Grade 5	5109 (9.7)	14,531 (11.0)

**Table 2:** Changes ^§^ of medians (P25–P75) in time of sedentary behaviors (SB) from 2002 to 2010–2012 (hours per day).

Variables	Total SB		Leisure Time SB		Occupational SB ^#^	
	2002	2010–2012	2002	2010–2012	2002	2010–2012
Total	3.0 (2.0–5.0)	3.0 (2.0–5.0) **	2.0 (1.5–3.0)	2.0 (2.0–3.0) **	4.0 (2.0–6.0)	4.0 (2.0–6.0) **
Gender						
Female	2.0 (1.5–4.0)	3.0 (2.0–5.0) **	2.0 (1.0–3.0)	2.0 (2.0–3.0) **	5.0 (2.5–6.0)	4.0 (2.0–6.0) **
Male	3.0 (2.0–6.0)	3.0 (2.0–6.0) **	2.5 (1.5–3.5)	2.5 (2.0–3.0) **	4.0 (2.0–6.0)	3.0 (2.0–6.0) **
Age group (year)						
18–29.9	3.5 (2.0–7.0)	4.0 (2.5–6.5) **	3.0 (2.0–4.0)	3.0 (2.0–4.0) **	4.0 (2.0–6.0)	4.0 (2.0–6.0) **
30–39.9	3.0 (2.0–6.0)	3.0 (2.0–6.0) **	2.0 (1.5–3.0)	2.0 (2.0–3.0) **	4.0 (2.5–6.0)	4.0 (2.0–6.0) **
40–49.9	3.0 (2.0–5.5)	3.0 (2.0–5.0) **	2.0 (1.5–3.0)	2.0 (2.0–3.0) **	4.0 (3.0–6.0)	4.0 (2.0–6.0) **
50–59.9	2.0 (1.5–4.0)	3.0 (2.0–4.0) **	2.0 (1.0–3.0)	2.0 (2.0–3.0) **	4.0 (3.0–6.0)	3.0 (2.0–6.0) **
≥60	2.0 (1.0–3.0)	2.5 (2.0–4.0) **	2.0 (1.0–3.0)	2.0 (2.0–4.0) **	4.6 (3.0–6.0)	3.0 (1.0–5.0) **
Region						
Urban	4.0 (2.5–7.5)	4.0 (2.0–7.0) **	3.0 (2.0–4.0)	3.0 (2.0–4.0) **	5.0 (2.0–6.0)	4.0 (2.0–6.0) **
Rural	2.0 (1.0–4.0)	3.0 (2.0–4.0) **	2.0 (1.0–3.0)	2.0 (2.0–3.0) **	6.0 (4.0–8.0)	5.0 (3.0–8.0) **
Occupation						
Employed	7.5 (5.0–10.0)	6.0 (4.0–9.0) **	3.0(2.0–4.0)	2.0 (2.0–3.0) **	4.0 (2.0–6.0)	4.0 (2.0–6.0) **
Farmers	2.0 (1.0–3.0)	2.0 (2.0–3.0) **	2.0 (1.0–3.0)	2.0 (2.0–3.0) **	/	/
Unemployed	2.5 (1.5–4.0)	3.0 (2.0–4.0) **	2.5 (1.5–4.0)	3.0 (2.0–4.0) **	/	/
Marital Status						
Yes	2.9 (2.0–5.0)	3.0 (2.0–5.0) **	2.0 (1.5–3.0)	2.0 (2.0–3.0) **	4.0 (2.0–6.0)	4.0 (2.0–6.0) **
No	3.0 (2.0–6.0)	4.0 (2.0–6.0) **	2.5 (1.5–4.0)	3.0 (2.0–4.0) **	4.0 (2.0–6.0)	4.0 (2.0–6.0) **
Educational Level						
Illiterate and primary school graduate	2.0 (1.0–3.0)	2.0 (2.0–4.0) **	2.0 (1.0–2.5)	2.0 (2.0–3.0) **	4.0 (1.5–6.0)	2.0 (1.0–5.0) **
Middle school graduate	3.0 (2.0–4.5)	3.0 (2.0–5.0) **	2.5 (2.0–3.0)	2.3 (2.0–3.0) **	4.0 (2.0–6.0)	3.0 (1.0–5.0) **
High school graduate or higher	6.0 (3.0–9.0)	5.0 (3.0–8.0) **	3.0(2.0–4.0)	3.0 (2.0–4.0) **	5.0 (3.0–6.0)	5.0 (3.0–6.0) **
Family economic level						
Grade 1	2.0 (1.0–3.0)	3.0 (2.0–4.0) **	2.0 (1.0–3.0)	2.0 (2.0–3.0) **	4.0 (2.0–6.0)	3.0 (1.0–5.0) **
Grade 2	2.0 (1.0–3.0)	3.0 (2.0–4.0) **	2.0 (1.0–3.0)	2.0 (2.0–3.0) **	4.0 (2.0–6.0)	3.0 (1.0–5.0) **
Grade 3	2.0 (1.0–3.0)	2.0 (1.5–3.0) **	2.0 (1.5–3.0)	2.5 (2.0–3.0) **	4.0 (2.0–6.0)	4.0 (2.0–6.0) **
Grade 4	4.5 (2.5–8.0)	4.0 (2.0–6.5) **	3.0 (2.0–4.0)	3.0 (2.0–4.0) **	4.0 (2.0–6.0)	4.0 (2.0–6.0) **
Grade 5	5.5 (3.0–9.0)	5.0 (3.0–8.0) **	3.0 (2.0–4.0)	3.0 (2.0–4.0) **	5.0 (3.0–6.0)	5.0 (3.0–7.0) **

^§^ gender and age standardized to the 2010 China census population; ^#^ Time of occupational SB was only calculated in employed participants, *n*2002 = 12,625, *n*2010–2012 = 31,914; ^**^ *p* < 0.001, significance is based on non-parametric tests.

**Table 3:** Trends ^§^ in the prevalence of total SB time ≥ 4 h/d, leisure SB time ≥ 3 h/d and occupational SB ^#^ time ≥ 4 h/d among Chinese adults from 2002 to 2010–2012.

Variables	Total SB Time ≥ 4 h/d				Leisure SB Time ≥ 3 h/d				Occupational SB Time ≥ 4 h/d			
	2002	2010–2012	Absolute Change (%)	Relative Change (%)	2002	2010–2012	Absolute Change (%)	Relative Change (%)	2002	2010–2012	Absolute Change (%)	Relative Change (%)
Total	35.4	43.0	+7.6 **	+21.5	42.0	48.0	+6.0 **	+14.3	63.4	53.0	−10.4 **	−16.4
Gender												
Female	29.8	38.3	+8.5**	+28.5	38.0	46.1	+8.1 **	+21.3	66.4	59.7	−6.7 **	−10.1
Male	40.8	47.6	+6.8**	+16.7	46.0	49.8	+3.8 **	+8.3	61.6	49.1	−12.5 **	−20.3
Age group (year)												
18–29.9	47.8	55.5	+7.7 **	+16.1	53.2	55.5	+2.3 *	+4.3	60.7	54.3	−6.4 **	−10.5
30–39.9	38.3	49.1	+10.8 **	+28.2	41.0	44.7	+3.7 **	+9.0	64.7	55.8	−8.9 **	−13.8
40–49.9	36.2	41.3	+5.1 **	+14.1	38.9	44.4	+5.5 **	+14.1	65.1	50.7	−14.4 **	−22.1
50–59.9	26.5	33.6	+7.1 **	+26.8	37.3	45.6	+8.3 **	+22.3	66.2	48.5	−17.7 **	−26.7
≥60	19.8	27.3	+7.5 **	+37.9	34.5	47.3	+12.8 **	+37.1	63.2	41.3	−21.9 **	−34.7
Region												
Urban	56.3	45.2	−11.1 **	−19.7	57.6	51.8	−5.8 **	−10.1	65.3	59.2	−6.1 **	−9.3
Rural	25.3	31.8	+6.5 **	+25.7	34.6	44.5	+9.9 **	+28.6	61.3	41.4	−19.9 **	−32.5
Occupation												
Employed	86.9	83.1	−3.8 **	−4.4	57.3	45.9	−11.4 **	−19.9	63.4	53.0	−10.4 **	−16.4
Farmers	9.6	14.4	+4.8 **	+50.0	28.7	40.8	+12.1 **	+42.2	/	/	/	/
Unemployed	27.6	30.5	+2.9 **	+10.5	47.5	54.7	+7.2 **	+15.2	/	/	/	/
Marital Status												
Yes	33.8	40.8	+7.0 **	+20.7	40.6	46.1	+5.5 **	+13.5	64.2	52.9	−11.3 **	−17.6
No	41.6	52.9	+11.3 **	+27.2	47.8	56.2	+8.4 **	+17.6	60.9	53.5	−7.4 **	−12.2
Educational Level												
Illiterate and primary school graduate	13.0	26.1	+13.1 **	+100.8	24.3	42.0	+17.7 **	+72.8	51.6	35.8	−15.8 **	−30.6
Middle school graduate	35.1	40.9	+5.8 **	+16.5	44.9	48.8	+3.9 **	+8.7	57.4	42.5	−14.9 **	−26.0
High school graduate or higher	71.1	65.7	−5.4 **	−7.6	65.5	53.8	−11.7 **	−17.9	69.3	65.3	−4.0 **	−5.8
Family economic level												
Grade 1	14.7	29.3	+14.6 **	+99.3	26.8	42.9	+16.1 **	+60.1	54.5	39.0	−15.5 **	−28.4
Grade 2	21.1	37.6	+16.5 **	+78.2	33.5	47.2	+13.7 **	+40.9	59.2	44.0	−15.2 **	−25.7
Grade 3	36.6	45.7	+9.1 **	+24.9	42.5	49.1	+6.6 **	+15.5	59.4	50.8	−8.6 **	−14.5
Grade 4	57.3	54.1	−3.2 **	−5.6	56.1	52.0	−4.1 **	−7.3	65.0	60.0	−5.0 **	−7.7
Grade 5	69.6	64.3	−5.3 **	−7.6	66.0	53.3	−12.7 *	−19.2	72.2	68.1	−4.1 **	−5.7

^§^ gender and age standardized to the 2010 China census population; ^#^ Time of occupational SB was only calculated in employed participants, *n*2002 = 12,625, *n*2010−2012 = 31,914; ** *p* < 0.001, significance is based on Chi−square tests.

**Table 4:** Correlates ^§^ of total SB time ≥ 4 h/d, leisure SB time ≥ 3 h/d, and occupational SB ^#^ time ≥ 4 h/d among Chinese adults in 2010–2012 (*n* = 131,560).

Independent Variables	Odds Ratio (95% Confidence Intervals)		
	Total SB Time ≥ 4 h/d	Leisure SB Time ≥ 3 h/d	Occupational SB ^#^ Time ≥ 4 h/d
Gender			
Female	1.0	1.0	1.0
Male	1.10 (1.10–1.11)	1.27 (1.27–1.28)	0.74 (0.74–0.74)
Age group (year)			
18–29.9	1.0	1.0	1.0
30–39.9	0.78 (0.77–0.78)	0.75 (0.75–0.75)	1.03 (1.03–1.04)
40–49.9	0.73 (0.73–0.73)	0.76 (0.76–0.76)	0.95 (0.95–0.96)
50–59.9	0.68 (0.68–0.69)	0.72 (0.72–0.73)	0.90 (0.89–0.90)
≥60	0.75 (0.75–0.75)	0.69 (0.68–0.69)	1.01 (1.00–1.01)
Region			
Rural	1.0	1.0	1.0
Urban	1.26 (1.26–1.27)	1.12 (1.12–1.12)	1.31 (1.30–1.31)
Occupation			
Unemployed	1.0	1.0	/
Farmers	0.52 (0.52–0.52)	0.65 (0.65–0.66)	/
Employed	9.56 (9.55–9.56)	0.55 (0.55–0.55)	/
Marital Status			
Yes	1.0	1.0	1.0
No	1.28 (1.27–1.28)	1.29 (1.28–1.29)	0.90 (0.89–0.90)
Educational Level			
Illiterate and primary school graduate	1.0	1.0	1.0
Middle school graduate	1.11 (1.10–1.11)	1.20 (1.19–1.20)	1.26 (1.25–1.26)
High school graduate or higher	1.53 (1.53–1.53)	1.35 (1.35–1.36)	2.50 (2.49–2.50)
Family economic level			
Grade 1	1.0	1.0	1.0
Grade 2	1.12 (1.11–1.12)	1.16 (1.16–1.16)	1.12 (1.14–1.15)
Grade 3	1.26 (1.26–1.27)	1.21 (1.20–1.21)	1.34 (1.33–1.34)
Grade 4	1.46 (1.45–1.46)	1.31 (1.30–1.31)	1.72 (1.71–1.72)
Grade 5	1.65 (1.64–1.65)	1.36 (1.36–1.37)	2.11 (2.11–2.12)

^§^ Gender and age standardized to the 2010 China census population; ^#^ Time of occupational SB was only calculated in employed participants, *n* = 31,914.

## Supplementary Materials

The following are available online at https://www.mdpi.com/1660-4601/17/1/158/s1, Table S1: Characteristics of the participants by gender and survey year [n (%)], Table S2: Changes (means) and absolute differences in time of sedentary behaviors (SB) from 2002 to 2010–2012 (hours per day), Table S3: Correlates of total SB time ≥ 4 h/d, leisure SB time ≥ 3 h/d, and occupational SB time ≥ 4 h/d among Chinese adults in 2010–2012 (economic missing cases included, *n* = 139,925).
