# Supplementary material for: Temporal Trends and Recent Correlates in Sedentary Behaviors among Chinese Adults from 2002 to 2010–2012
Source: Int J Environ Res Public Health. 2019 Dec 24;17(1):158. doi: 10.3390/ijerph17010158 (PMC6982214; doi:10.3390/ijerph17010158)
Supplement: Supplementary file 1 [file ijerph-17-00158-s001.pdf]

Supplementary materials

Table S1. Characteristics of the sample by gender and survey year [*n* (%)].

Variables	2002		2012	
	**Male**	**Female**	**Male**	**Female**
Age group(year)				
18–29.9	3618 (14.5)	4376 (15.8)	5006 (8.6)	8814 (12.0)
30–39.9	5935 (23.8)	6999 (25.2)	7889 (13.5)	10,890 (14.9)
40–49.9	5466 (21.9)	6188 (22.3)	13,064 (22.4)	16,746 (22.9)
50–59.9	4934 (19.8)	5261 (19.0)	13,806 (23.7)	17,092 (23.3)
≥60	5007 (20.1)	4913 (17.7)	18,513 (31.8)	19,740 (26.9)
Region				
Urban	8148 (32.6)	9310 (33.6)	28,214 (48.4)	37,005 (50.5)
Rural	16,812 (67.4)	18,427 (66.4)	30,064 (51.6)	36,277 (49.5)
Occupation				
Employed	7581 (30.4)	5044 (18.2)	18,183 (31.2)	13,731 (18.7)
Farmers	11,890 (47.6)	10,911 (39.3)	18,862 (32.4)	19,855 (27.1)
Unemployed	5489 (22.0)	11,782 (42.5)	21,233 (36.4)	39,696 (54.2)
Marital Status				
Yes	21,920 (87.8)	23,969 (86.4)	52,233 (89.6)	63,169 (86.2)
No	3040 (12.2)	3768 (13.6)	6045 (10.4)	10,113 (13.8)
Educational Level				
Illiterate and primary school graduate	8433 (33.8)	13,834 (49.9)	20,232 (34.7)	34,103 (46.5)
Middle school graduate	9825 (39.4)	8609 (31.0)	22,674 (38.9)	23,093 (31.5)
High school graduate or higher	6702 (26.8)	5294 (19.1)	15,372 (26.4)	16,086 (22.0)
Family's economic level				
Grade 1	3577 (14.3)	3985 (14.4)	15,900 (27.3)	19,598 (26.7)
Grade 2	7423 (29.7)	8258 (29.8)	14,399 (24.7)	17,982 (24.5)
Grade 3	7224 (28.9)	8071 (29.1)	11,114 (19.1)	14,111 (19.3)
Grade 4	4311 (17.3)	4739 (17.1)	10,542 (18.1)	13,383 (18.3)
Grade 5	2425 (9.7)	2684 (9.7)	6323 (10.8)	8208 (11.2)
Total	24,960 (100.0)	27,737 (100.0)	58,278 (100.0)	73,282 (100.0)

Table S2. Changes § (means) and absolute differences in time of sedentary behaviors (SB) from 2002 to 2010–2012 (hours per day).

Variables	Total SB			Leisure Time SB			Occupational SB #		
	2002	2010–2012	D ##	2002	2010–2012	D ##	2002	2010–2012	D ##
Total	3.71	4.01	+0.29 **	2.57	2.71	+0.14 **	4.41	4.02	−0.39 **
Gender									
Female	3.32	3.72	+0.41 **	2.40	2.67	+0.27 **	4.58	4.34	−0.24 **
Male	4.10	4.28	+0.18**	2.73	2.75	+0.01 **	4.30	3.83	−0.48 **
Age group(year)									
18–29.9	4.66	4.78	+0.12 **	3.03	2.92	−0.11 **	4.32	4.15	−0.17 **
30–39.9	4.05	4.47	+0.42 **	2.52	2.60	+0.08 **	4.50	4.14	−0.36 **
40–49.9	3.85	3.94	+0.09 **	2.47	2.57	+0.10 **	4.43	3.86	−0.57 **
50–59.9	3.04	3.42	+0.38 **	2.40	2.64	+0.24 **	4.40	3.69	−0.71 **
≥60	2.30	2.88	+0.58 **	2.20	2.75	+0.56 **	4.64	3.33	−1.30 **
Region									
Urban	5.11	4.76	−0.35 **	3.24	2.88	−0.36 **	4.47	4.29	−0.18 **
Rural	3.04	3.31	+0.26 **	2.25	2.55	+0.30 **	4.34	3.51	−0.83 **
Occupation									
Employed	7.52	6.61	−0.91 **	3.14	2.59	−0.54 **	4.41	4.02	−0.39 **
Farmers	2.04	2.40	+0.36 **	2.04	2.40	+0.36 **	/	/	/
Unemployed	2.81	3.01	+0.20 **	2.81	3.01	+0.20 **	/	/	/
Marital Status									
Yes	3.62	3.88	+0.26 **	2.50	2.65	+0.14 **	4.44	3.99	−0.44 **
No	4.09	4.59	+0.49 **	2.83	2.98	+0.16 **	4.32	4.10	−0.22 *
Educational Level									
Illiterate and primary school graduate	2.15	2.93	+0.79 **	1.87	2.53	+0.66 **	3.97	3.21	−0.76 **
Middle school graduate	3.66	3.82	+0.16 **	2.66	2.72	+0.06 **	4.18	3.57	−0.62 **
High school graduate or higher	6.26	5.51	−0.74 **	3.52	2.90	−0.62 **	4.63	4.56	−0.07 **
Family's economic level									
Grade 1	2.19	3.14	+0.95 **	1.93	2.53	+0.59 **	3.85	3.37	−0.48 **
Grade 2	2.75	3.65	+0.89 **	2.23	2.66	+0.43 **	4.28	3.57	−0.71 **
Grade 3	3.78	4.14	+0.35 **	2.57	2.76	+0.19 **	4.28	3.89	−0.39 **
Grade 4	5.26	4.69	−0.57 **	3.14	2.85	−0.29 **	4.40	4.35	−0.05 **
Grade 5	6.14	5.49	−0.65 **	3.59	2.91	−0.68 **	4.84	4.75	−0.09 **

§ gender and age standardized to the 2010 China census population; # Time of Occupational SB was only calculated in employed participants, *n*2002 = 12,625, *n*2010–2012 = 31,914; ## D for Difference; + = increase; − = decrease; ** $p < 0.001$, * $p < 0.05$, significance is based on T-Tests.

Table S3. Correlates § of total SB time ≥ 4 h/d, leisure SB time ≥ 3 h/d, and occupational SB # time ≥ 4 h/d among Chinese adults in 2010–2012 (economic missing cases included, *n* = 139,925).

Independent Variables	OR (95% CI)		
	Total SB time ≥ 4 h/d	Leisure SB time ≥ 3 h/d	Occupational SB # time ≥ 4 h/d
Gender			
Female	1.0	1.0	1.0
Male	1.11 (1.10–1.11)	1.27 (1.27–1.28)	0.74 (0.74–0.74)
Age group (year)			
18–29.9	1.0	1.0	1.0
30–39.9	0.78 (0.77–0.78)	0.75 (0.74–0.75)	1.03 (1.03–1.03)
40–49.9	0.73 (0.73–0.74)	0.76 (0.76–0.76)	0.95 (0.95–0.95)
50–59.9	0.68 (0.68–0.68)	0.72 (0.72–0.72)	0.90 (0.89–0.90)
≥60	0.75 (0.75–0.75)	0.69 (0.68–0.69)	1.01 (1.00–1.01)
Region			
Rural	1.0	1.0	1.0
Urban	1.26 (1.26–1.26)	1.12 (1.12–1.12)	1.32 (1.31–1.32)
Occupation			
Unemployed	1.0	1.0	/
Farmers	0.52 (0.52–0.52)	0.65 (0.65–0.66)	/
Employed	9.64 (9.63–9.64)	0.55 (0.55–0.55)	/
Marital Status			
Yes	1.0	1.0	1.0
No	1.28 (1.27–1.28)	1.29 (1.28–1.29)	0.90 (0.89–0.90)
Educational Level			
Illiterate and primary school graduate	1.0	1.0	1.0
Middle school graduate	1.11 (1.10–1.11)	1.20 (1.19–1.20)	1.26 (1.25–1.26)
High school graduate or higher	1.53 (1.53–1.53)	1.35 (1.35–1.36)	2.50 (2.49–2.50)
Family's economic level			
Grade 1	1.0	1.0	1.0
Grade 2	1.12 (1.11–1.12)	1.16 (1.16–1.16)	1.15 (1.14–1.15)
Grade 3	1.26 (1.26–1.27)	1.21 (1.20–1.21)	1.34 (1.33–1.34)
Grade 4	1.46 (1.45–1.46)	1.31 (1.30–1.31)	1.71 (1.71–1.72)
Grade 5	1.64 (1.64–1.65)	1.36 (1.36–1.36)	2.11 (2.11–2.12)
Unknown	1.19 (1.19–1.20)	1.09 (1.09–1.09)	1.74 (1.73–1.74)

§ gender and age standardized to the 2010 China census population; # Time of occupational SB was only calculated in employed participants, *n* = 34,394.
